# Supplementary material for: Influence of tied-ridge-furrow with inorganic fertilizer on grain yield across semiarid regions of Asia and Africa: A meta-analysis
Source: PeerJ. 2021 Aug 17;9:e11904. doi: 10.7717/peerj.11904 (PMC8378338; doi:10.7717/peerj.11904)
Supplement: Supplemental Information 3 [file peerj-09-11904-s003.docx]

1. Rationale for conducting the meta-analysis

In rain-fed agro-ecological areas in Asia and Africa, the effects of fertilizer in tied-ridge-furrow on crop yield, WUE, and soil physical properties have been recorded in the past with mixed results. Therefore, we conducted a meta-analysis on related literature to determine the effect of inorganic fertilizer in tied-ridge-furrow on grain yield and soil physical properties.

2. The contribution that it makes to knowledge in light of previously published related reports including other meta-analysis

This meta-analysis will bring to light understanding of how plant biomass worldwide, particularly in Asia and Africa change with the application of fertilizers and mulching in tied ridges and also to harnessing their potential for large-scale implementation in agricultural production
